# Two cases of pancytopenia characterized by severe neutropenia induced by anti-PD-1 tislelizumab combined with chemotherapy in non-small cell lung cancer patients and a literature review

**DOI:** 10.3389/fmed.2026.1820443

**Published:** 2026-07-06

**Authors:** Fang Lu, Yi Li, Yaqian Zhu, Wenkang Zong, Fang Zhao, Yongxiang Zhang, Liheng Yang, Yuechuan Li

**Affiliations:** 1 Tianjin Chest Hospital, Tianjin University, Tianjin, China; 2 Department of Respiratory and Critical Care Medicine, Tianjin Chest Hospital, Tianjin, China; 3 Department of Pathology, Tianjin Chest Hospital, Tianjin, China

**Keywords:** immune checkpoint inhibitors, immune-related adverse events, lung cancer, neutropenia, pancytopenia, tislelizumab

## Abstract

**Background:**

With the increasing use of immune checkpoint inhibitors (ICI) in clinical practice of lung cancer, more immune-related adverse events (irAEs) are being discovered. However, hematologic irAEs (hem-irAEs) were rarely described.

**Case presentation:**

Two patients with non-small cell lung cancer were treated with tislelizumab and chemotherapy as the first-line treatment. Immune-related pancytopenia (grade 4 neutropenia, grade 3–4 thrombocytopenia, and grade 3 anemia) occurred after the 3rd (Day 144) and 2nd (Day 32) cycles, respectively. Case 1 had concomitant immune-related hepatitis (grade 3) while Case 2 presented with immune-related mucositis and renal insufficiency (grade 2). Bone marrow examination of Case 1 revealed a hypocellular marrow with increased mature lymphocytes. The patients were given granulocyte-stimulating factor (G-CSF), thrombopoietin, component transfusion (platelet-rich buffy coat and apheresis platelets), and corticosteroids (0.72–1.64 mg/kg/d). Grade 4 neutropenia persisted, with only mild improvements in platelets and red blood cells. Intermittent fever and pulmonary infection were managed with antibacterial and antifungal therapy. Regrettably, both patients died of multiple organ dysfunction syndrome. Naranjo Scale rated tislelizumab-induced pancytopenia as possible in both cases.

**Conclusion:**

Although hem-irAEs are rare, they should be distinguished from chemotherapy-induced myelosuppression. Clinicians should remain vigilant, monitor blood cell counts regularly, and intervene promptly to prevent severe complications.

## 1 Introduction

Immune checkpoint inhibitors therapy has changed the landscape of managing multiple cancer types in the last decade. These immunomodulatory monoclonal antibodies block cytotoxic T-lymphocytes–associated antigen 4 (CTLA-4) and programmed cell death protein 1 (PD-1) or its ligand (PD-L1), thus restoring or augmenting an antitumor immune response (1, 2). However, as a consequence of immune system activation, a broad range of irAEs may arise (1–3). The most frequent irAEs include dermatitis, pneumonitis, hypothyroidism, colitis, liver dysfunction, while less frequent events refer to myalgias and arthralgias, hypophysitis (4–6), and hematological side effects (7, 8). Hematologic irAEs (hem-irAEs) are rare following ICI treatment, some reported less than 1% (7), some reported with a frequency of 3.6% for all grades and a mortality rate reported to be 14% (8), which include immune thrombocytopenia (ITP), immune-related neutropenia (irN), aplastic anemia (AA)/pancytopenia, autoimmune hemolytic anemia (AIHA), and hemophagocytic syndrome (HPS) (7). Presenting with multilineage cytopenia, it is associated with numerous complications and a high mortality rate. Although the prognosis of hematological irAE is poor, the management is not well-established, especially irN and immune thrombocytopenia (ITP). The recommendations for the management of irAEs published earlier by Naidoo do not mention irN (9), whereas European Society for Medical Oncology (ESMO) (10) guidelines address the hematological toxicities as a group without specifying the optimal management. American Society of Clinical Oncology (ASCO) guidelines address several other hematological toxicities (3), but not irN. Nevertheless, given the increasingly widespread and rapid adoption of ICI therapies across various malignancies, irAEs, particularly irN, are likely to manifest in larger patient populations. Differentiation is particularly challenging in cases where chemotherapy is administered concurrently. Therefore, we should pay more attention on these fatal side-effects of ICI and develop more efficacy strategies. Here, we report two cases of hematological adverse events induced by tislelizumab in patients with non-small cell lung cancer (NSCLC).

## 2 Case presentation

### 2.1 Case 1

**FIGURE 1:**
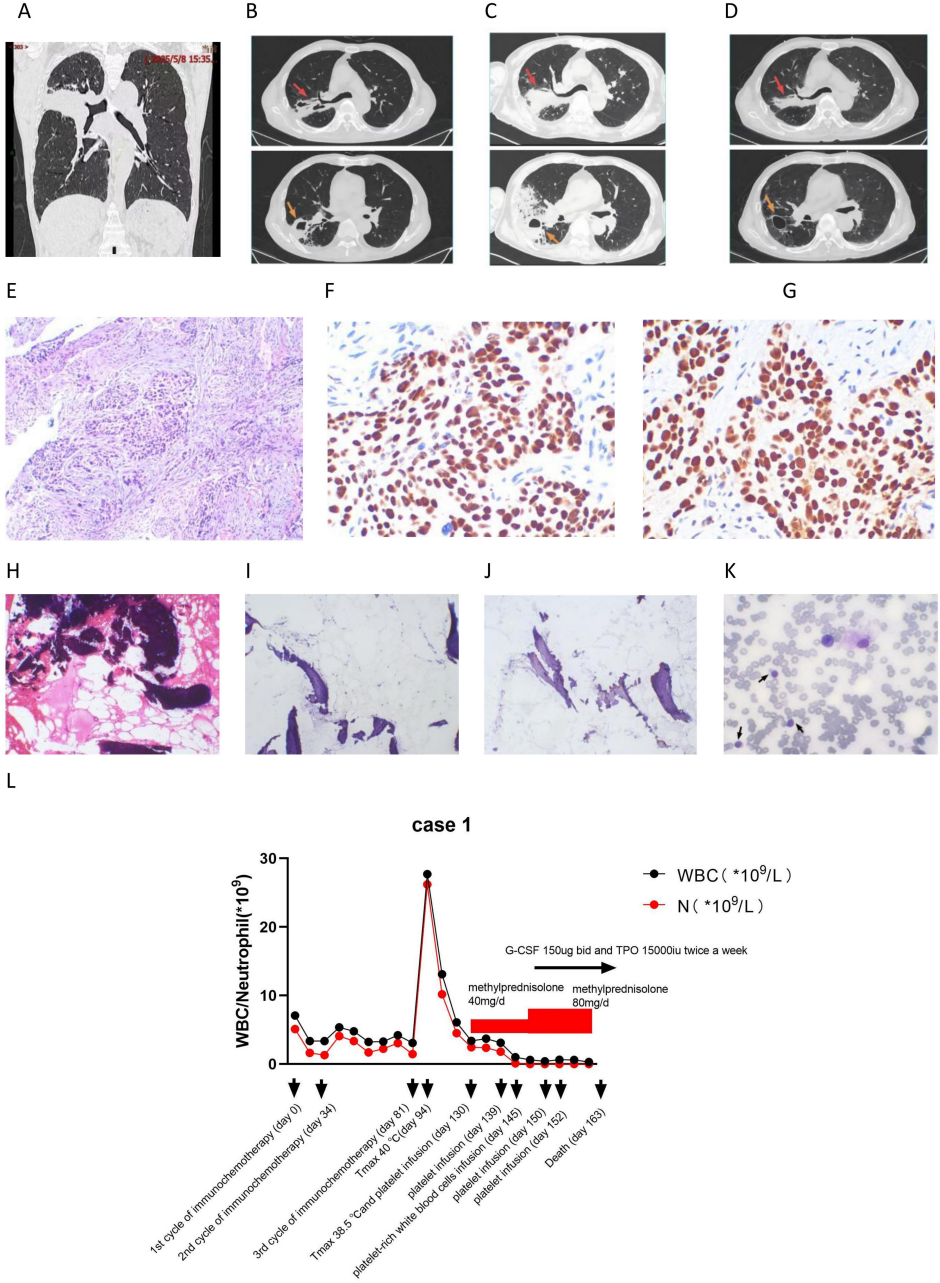
**(A–D)** Chest high-resolution computed tomography. **(A)** 2025-05-08 (coronal): right hilar mass. **(B)** 2025-07-18 (after 2 cycles): primary lesion decreased; new cavity in right lower lung. **(C)** 2025-08-16 (axial, after three cycles): primary and infectious lesions enlarged. **(D)** 2025-09-26 (axial, after anti-infective therapy): Both lesions decreased. (red arrow: primary lesion, yellow arrow: infectious lesion). **(E–G)** Bronchoscopic biopsy pathology. **(E)** HE staining (×100). **(F)** IHC P40 (+) (×400). **(G)** IHC P53 (+) (×400); **(H–J)** Iliac bone biopsy (Tianjin First Central Hospital, 23 Oct). **(H)** HE staining (×100): Empty bone marrow with scattered rare nucleated cells; no megakaryocytes or carcinoma cells detected. **(I)** IHC CK7 (–). **(J)** IHC P40 (–). **(K)** Bone marrow smear (Tianjin First Central Hospital, Oct 23) (×400): markedly increased proportion of lymphocytes (mature lymphocytes, black arrow). **(L)** Dynamic changes in absolute leukocyte and neutrophil counts after immunochemotherapy.

A 68-year-old non-smoking male presented to Tianjin Chest Hospital with two months of intermittent cough and expectoration, and had a history of seborrheic dermatitis. Chest CT showed a right upper lobe mass with partial bronchial obstruction and suspicious metastatic lymphadenopathy (Figure 1A). Bronchoscopy confirmed poorly differentiated right upper lobe squamous cell carcinoma (stage IIIB, T3N2bM0, AJCC 8th edition) combined with chronic obstructive pulmonary disease (Figures 1E–G).

After thoracic surgery consultation, the tumor was judged potentially resectable. The patient started neoadjuvant tislelizumab 200 mg combined with liposomal paclitaxel 240 mg (134.8 mg/m^2^, BSA 1.78 m^2^) and carboplatin 0.4 g (AUC = 3.8 mg⋅mL^–1^min^–1^) on 17 May 2025. Baseline blood routine, cardiac, hepatic and renal function were normal except mild anemia (Hb 98 g/L). On Day 26, he developed grade 3 immune-related hepatitis with ALT 476.5 and AST 411.1 U/L, which resolved after bicyclol and methylprednisolone (prednisone equivalent 0.72 mg/kg/d). Grade 1 leukopenia (neutrophils 1.31 × 10^9^/L) occurred on Day 33. After granulocyte colony-stimulating factor (G-CSF) intervention, he received chemotherapy alone with immunotherapy suspended. On Day 62, he presented with fatigue, cough and dyspnea. Chest CT showed partial tumor regression, as well as a new thick-walled cavity and surrounding patchy opacities in the right lower lobe (Figure 1B), which improved after cefoperazone-sulbactam plus voriconazole.

Follow-up revealed enlarged right hilar lesions. Although most guidelines (SITC; ASCO; NCCN; ESMO) recommended permanent discontinuation of immunotherapy for patients with severe hepatotoxicity of grade 3 or higher, the 2019 CSCO guidelines (11) suggested that patients with grade 3 hepatotoxicity may resume immunotherapy once hepatic function recovers to grade 1 and glucocorticoids is tapered to a prednisone-equivalent dose of 10 mg, with only grade 4 cases requiring permanent discontinuation. With liver function fully normalized for 1 month and informed family consent, immunotherapy was resumed. The patient (ECOG PS 2) received tislelizumab 200 mg plus single-agent liposomal paclitaxel 240 mg on 6 August 2025 (Day 81). He later developed hyperpyrexia up to 40 °C, hypotension and impaired consciousness with progressive pulmonary lesions (Figure 1C). Recurrent liver injury occurred on Day 94 (ALT 570.8, AST 348.3 U/L). He was given anti-infection drugs, methylprednisolone (0.72 mg/kg/d), norepinephrine and supportive treatment, and finally discharged after clinical improvement.

On Day 130, the patient was readmitted due to fever (T_*max*_ 38.5°C) and hemoptysis. Tests showed grade 4 thrombocytopenia (23 × 10^9^/L), cardiac insufficiency (LVEF 35%–40%) and CRP 80.2 mg/L. Chest CT on Day 132 (26 September 2025) showed shrunk right upper lobe lesion and improved right lower lobe lesions (Figure 1D), and immune-related myelotoxicity and cardiotoxicity were suspected. He was given meropenem, subcutaneous TPO, apheresis platelet transfusion and intravenous methylprednisolone 20 mg/d (prednisone 0.36 mg/kg/d). Platelets dropped to 10 × 10^9^/L on Day 137 and rose to 52 × 10^9^/L on Day 144. Meanwhile, grade 4 neutropenia (WBC 1.02 × 10^9^/L, N 0.12 × 10^9^/L) and grade 3 anemia (Hb 77 g/L) occurred. Subsequently, G- CSF 150 μg was administered twice daily, along with blood transfusion and intravenous methylprednisolone 40–80 mg/d (prednisone 0.72–1.44 mg/kg/d). However, persistent grade 4 neutropenia remained (WBC 0.62 × 10^9^/L, N 0.02 × 10^9^/L), accompanied by Hb 66 g/L and PLT 15–34 × 10^9^/L.

On Day 159 (23 October 2025), the patient was transferred for bone marrow aspiration and iliac biopsy (Figures 1H–J). Pathological findings revealed severe hematopoietic hypoplasia, marked adipose replacement and medullary hemorrhage, with scarce nucleated cells and absence of megakaryocytes and malignant cells. Immunohistochemistry showed scattered positive MPO, CD15, CD235a, CD163 and negative CD42b, CK, CK7, TTF-1, p40, CD138, with reticular fiber grade MF = 0. Bone marrow smear indicated obviously elevated mature lymphocytes (Figure 1K). The patient suffered intractable severe myelosuppression: WBC 0.32 × 10^9^/L, N 0 × 10^9^/L, Hb 64 g/L, PLT 5 × 10^9^/L. Concurrent complications included fungal infection, sepsis, cardiac insufficiency and hepatorenal damage. Relevant indicators: GM 8.52 μg/L, CRP 216.54 mg/L, PCT 32.2 ng/mL, Pseudomonas aeruginosa PCR 1,528 copies/mL, NT-proBNP > 35,000 pg/mL, serum creatinine 129.3 μmol/L, total bilirubin 187.95 μmol/L. The patient died of multiple organ failure on Day 163 (October 27). Dynamic hematological alterations are shown in Figure 1L.

### 2.2 Case 2

**FIGURE 2:**
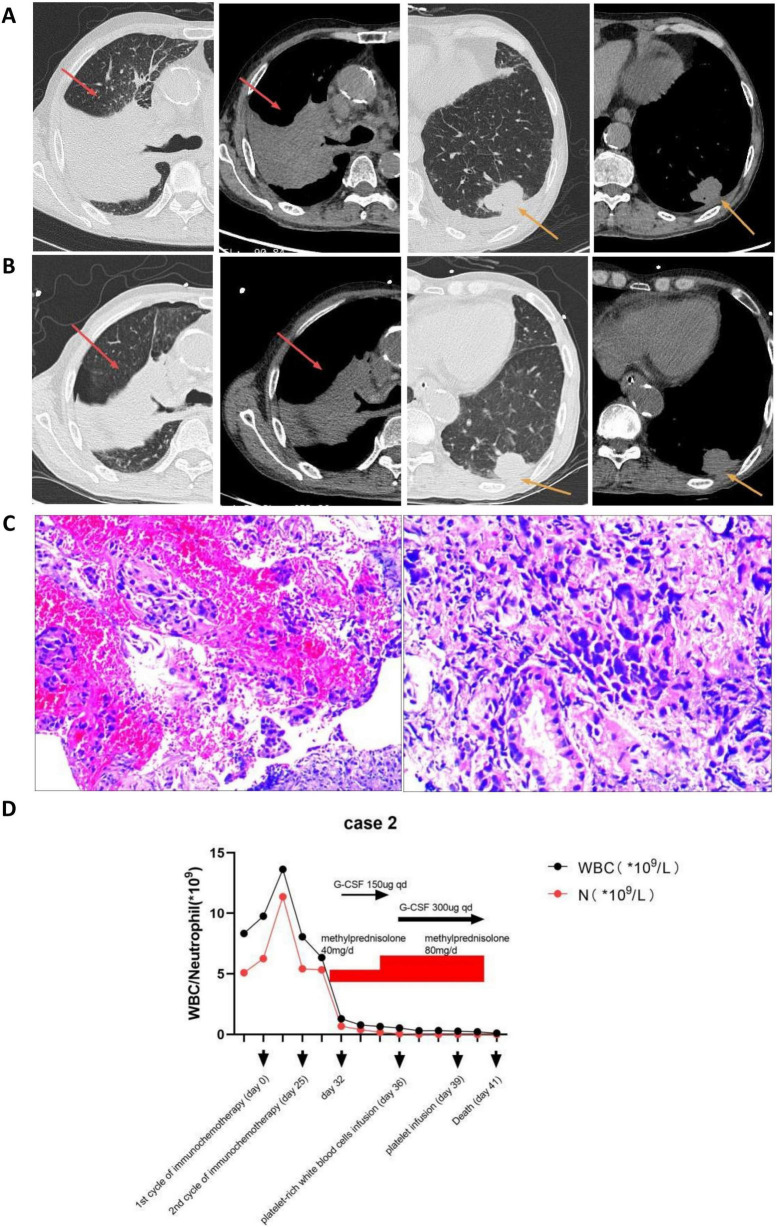
The CT imaging of lung cancer in superior lobe of right lung with metastatic carcinoma in inferior lobe of left lung **(A)**. The change of lung cancer after second cycle of immunochemotherapy in CT **(B)**. Histopathological characteristics of percutaneous lung biopsy **(C)**. The dynamic change in absolute count of white blood cell and neutrophil after immunochemotherapy **(D)**.

A 73-year-old male with hypertension (no smoking/tumor family history) presented to Tianjin Chest Hospital with a 1-month interrupted cough. Physical examination showed slightly diminished breath sounds in the right lung. Contrast-enhanced chest CT revealed a right hilar tumor with obstructive atelectasis and a left lower lobe nodule (Figure 2A). Bronchoscopy and percutaneous lung biopsy confirmed adenocarcinoma in both lesions, leading to the diagnosis of metastatic right upper lung adenocarcinoma [driver gene-negative, T4N3M1 (left lung metastasis)] (Figure 2C). Baseline blood routine (WBC, neutrophil, Hb, PLT) was normal (Table 1). The first cycle of tislelizumab 200 mg plus pemetrexed disodium 0.8 g (494 mg/m^2^, BSA 1.62 m^2^) and carboplatin 0.4 g (AUC 5 mg/mL/min) was administered from 19 to 22 September 2023 (Day 0–4), and the patient was discharged uneventfully. After the second cycle of immunochemotherapy on 17 October 2023 (Day 28), intermittent diarrhea occurred. On Day 29, blood routine showed grade 2 anemia (Hb 97 g/L), and antidiarrheal (montmorillonite powder 3 g tid) plus fluid therapy was given. On Day 30 (19 October 2023), renal insufficiency developed (serum creatinine 236 μmol/L); suspected immune-related grade 2 renal insufficiency, intravenous methylprednisolone 40 mg qd (prednisone 0.82 mg/kg/d) was initiated.

**TABLE 1:** Timeline of blood cell counts and biochemical parameters.

Case	Date	Days of ICI treatment	WBC (10^9^/L)	N (10^9^/L)	L (10^9^/L)	Hb (g/L)	PLT (10^9^/L)	ALT (U/L)	AST (U/L)	BUN (mmol/L)	Cr (μmol/L)	CRP (mg/L)	PCT (ng/mL)	LDH (U/L)
Case 1	2025-05-08	−9	7.1	5.11	1.29	98	464	7.9	9.2	6.5	75.02	60.1	0.06	209
	2025-05-17	0	–	–	–	–	–	–	–	–	–	–	–	–
	2025-05-20	3	7.61	6.66	0.78	105	348	–	–	6.7	68.52	–	–	–
	2025-06-12	26	3.35	1.65	1.32	110	141	476.5	411.1	–	–	6.04		566
	2025-06-19	33	3.35	1.31	1.19	104	254	227.4	157.2	–	–	–	–	–
	2025-06-21	35	5.36	4.09	1.06	99	233	145.5	142	–	–	–	–	–
	2025-07-09	53	4.78	3.35	1.08	91	283	7.8	11.8	–	–	16.7	–	–
	2025-07-19	63	4.22	3.07	0.81	92	205	–	–	–	–	–	–	–
	2025-08-06	81	3.08	1.47	1.22	105	266	7.6	16.1	4.9	55.39	3.98	–	168
	2025-08-19	94	27.71	26.2	0.72	111	62	570.8	348.3	27.7	178.3	125	–	714
	2025-08-27	102	6.1	4.5	1.11	101	151	33.4	17.5	4.5	57.84	18.6	2.85	315
	2025-09-24	130	3.39	2.46	0.72	101	23	43.3	34.3	5.9	68.65	80.2	0.75	254
	2025-10-01	137	3.1	1.8	1.24	90	10	17.9	13.9	8.5	54.06	–	–	–
	2025-10-08	144	1.02	0.12	0.89	77	52	26.1	15.2	5.4	43.09	–	–	–
	2025-10-13	149	0.61	0.02	0.58	66	24	17.7	7.6	7.2	45.42	–	–	–
	2025-10-14	150	0.4	0.01	0.38	73	15	–	–	–	–	–	–	–
	2025-10-16	152	0.63	0.02	0.56	66	34	–	–	–	–	–	–	–
	2025-10-18	154	0.62	0.02	0.58	66	75	–	–	–	–	–	–	–
	2025-10.26	162	0.32	0	0	64	5	7.2	10.9	15.58	129.3	216.54	32.26	–
	2025-10.27	163	–	–	Death	–	–	–	–	–	–	–	–	–
Case 2	2023-08-29	−21	8.33	5.1	2.27	128	381	7.7	10.4	5.4	90	16.1	–	236
	2023-09-19	0	9.75	6.26	1.99	133	428	11.1	9.5	10	106	9.87	–	267
	2023-09-23	4	13.64	11.38	1.39	121	391	–	–	–	–	–	–	–
	2023-10-14	25	8.06	5.42	1.58	102	634	23.7	20.6	9.7	149	6.52		308
	2023-10-18	29	6.34	5.33	0.59	97	535	–	–	–	–	–	–	–
	2023-10-19	30	–	–	–	–	–	–	17.9	16.8	236	–	–	312
	2023-10-20	31	–	–	–	–	–	–	–	17.8	256	–	–	–
	2023-10-21	32	1.29	0.68	0.52	87	397	–	–	17.7	220	–	–	–
	2023-10-22	33	0.78	0.39	0.37	93	341	–	–	16.8	198	–	–	–
	2023-10-23	34	0.66	0.18	0.44	92	253	–	–	15.6	184	–	–	–
	2023-10-24	35	0.53	0.05	0.46	87	182	–	–	15.1	161	–	–	–
	2023-10-25	36	0.31	0.02	0.28	87	155	12.9	14.5	149	16.6	145	0.44	168
	2023-10-26	37	0.32	0.01	0.31	95	99	53	30.2	127	18.2	–	–	–
	2023-10-27	38	0.27	0	0.26	89	56	–	–	108	18.7	–	–	131
	2023-10-28	39	0.22	0.01	0.21	84	27	–	–	–	–	–	–	–
	2023-10-29	40	0.11	0	0.11	79	39	189.3	98.7	16.2	101	184.3	–	161
	2023-10-30	41	–	–	Death	–	–	–	–	–	–	–	–	–

ICI, immune checkpoint inhibitor; WBC, white blood cells; N, neutrophil; L, lymphocyte; Hb, hemoglobin; PLT, platelets; ALT, alanine aminotransferase; AST, aspartate aminotransferase; BUN, blood urea nitrogen; Cr, creatinine; CRP, C-reactive protein; PCT, Procalcitonin; LDH, lactate dehydrogenase.

On 21 October 2023 (Day 32), blood routine showed grade 3 neutropenia (WBC 1.29 × 10^9^/L, N 0.68 × 10^9^/L) and serum creatinine 220 μmol/L (Table 1). He received G-CSF 150 μg qd for 3 days, but WBC and neutrophils continued to decline, progressing to grade 4 neutropenia (WBC 0.78 × 10^9^/L, N 0.39 × 10^9^/L) on Day 33, with urgent hematology consultation. Fever occurred, and sputum culture isolated *Acinetobacter baumannii*; meropenem 1 g q12h was added for anti-infection. On Day 35 (24 October), blood routine showed WBC 0.53 × 10^9^/L, N 0.05 × 10^9^/L, and he received six units of platelet-rich leukocyte suspension. Hematology consultation suggested possible hem-irAEs: methylprednisolone was continued, G-CSF was increased to 300 μg bid, and voriconazole was recommended for antifungal prophylaxis (Figure 2D). Bone marrow examination was advised but refused by the patient due to critical condition. On Day 36 (25 October), serum creatinine decreased to 164 μmol/L, but leukopenia persisted (WBC 0.31 × 10^9^/L, N 0.02 × 10^9^/L); methylprednisolone was increased to 80 mg qd (prednisone 1.64 mg/kg/d). The patient had intermittent high fever (T_max_ 40°C), a CRP of 145 mg/L, positive (1, 3)-β-D-glucan (550.9 pg/ml), and a positive GM test (1.22). Repeat chest CT showed partial resolution of obstructive atelectasis and no significant change in the metastatic lesions in the left lung (Figure 2B). So, oral voriconazole 200 mg q12h was given. Severe diarrhea, oral ulcers and poor intake occurred, with nasogastric tube placement for enteral nutrition support.

Starting on 26 October (Day 37), platelet count began to decrease (99 × 10^9^/L). Blood routine showed grade 3 thrombocytopenia (56 × 10^9^/L on Day 38, 27 × 10^9^/L on Day 39). Despite the transfusion of one therapeutic dose of apheresis platelets, which raised the platelet count to 39 × 10^9^/L, grade 4 neutropenia persisted (N: 0–0.01 × 10^9^/L from 26 to 29 October), and sputum culture again isolated *A. baumannii*. The patient remained critically ill with persistent fever (38–39°C from 28 October). On 29 October, multiple organ dysfunction occurred: impaired sputum expectoration, hypoxemia (oxygenation index < 150), oliguria, and hypotension (85–90/54–60 mmHg). Laboratory results: ALT 189.3 U/L, serum creatinine 101 μmol/L, BUN 16.2 mmol/L, CRP 184.3 mg/L. He was admitted to RICU and received invasive mechanical ventilation for progressive respiratory failure. The family subsequently requested discharge against medical advice. A follow-up confirmed his death locally on 30 October.

## 3 Discussion

The estimated incidence of hem-irAEs ranges from 0.5% to 3.6% (7, 8, 12), however, their severity is relatively high, with grade 4 and above accounting for 44-77% (7, 13). The frequency of Hem-irAEs was found to be lower with anti-CTLA-4 (0.5%) than with anti-PD1 (4.1%) or anti-PD-L1 (4.7%) (8), but anti-CTLA-4-based therapy occurred much earlier (median 23 vs. 47.5 days, *p* = 0.006) (14). ITP and AHA represent the most common hematological irAEs (13, 14). All- grade neutropenia occurs in 0.18% (14/7626)–0.95% (9/948) of all ICI - treated patients (7, 12, 15), with a febrile neutropenia rate of 0.45% (15). Meanwhile, pancytopenia or aplastic anemia constitutes 0.53% (5/948) (7). Febrile irN and pancytopenia typically present with severe clinical features and confer a dismal prognosis. It is noteworthy that these case series studies and systematic reviews involve a small number of cases, and only a portion of them are lung cancer cases (26.3%–34%) (7, 13). In addition, most reported cases of hem-irAEs are associated with nivolumab, pembrolizumab, ipilimumab, and atezolizumab, and most cases involved ICI monotherapy. Herein, we summarized 25 published cases of hem-irNs in lung cancer patients (Table 2), representing a relatively large sample size in relevant studies (7, 16–31). Only five cases were treated with chemoimmunotherapy. Two of them presented with grade 4 neutropenia and grade 2–4 thrombocytopenia, and no cases of pancytopenia were identified. Only one relevant case related to tislelizumab was retrieved in our literature search. In 2025, Zhou et al. reported a 75-year-old male who developed grade 4 irN lasting 20 days complicated with toxic epidermal necrolysis after tislelizumab plus chemotherapy, and improved following glucocorticoid and intravenous immunoglobulins (IVIG) treatment. Both patients in our study developed pancytopenia after treatment with tislelizumab plus chemotherapy, predominantly characterized by persistent neutropenia.

**TABLE 2:** Summary of cases with immune-related neutropenia in lung cancer patients.

Patients no.[ref.]	Age	Sex	Diagnosis	Medical history	ICPi agent	No. of ICPi cycles before onset (days after ICPi)	Neutropenia severity	Other ir-AEs (onset days post ICPi)
1 (7)	61	F	NSCLC	N	Nivolumab 240 mg every 2 weeks	6 (82)	Grade 4	N/A
2 (7)	52	F	NSCLC	N	Nivolumab 240 mg every 2 weeks	23 (332)	Grade 2	N/A
3 (7)	73	F	NSCLC	N	Nivolumab 240 mg every 2 weeks	4 (60)	Grade 4	N/A
4 (7)	78	M	NSCLC	N	Nivolumab 240 mg every 2 weeks	1 (14)	Grade 4	N/A
5 (7)	70	M	NSCLC	N	Nivolumab 240 mg every 2 weeks	6 (152)	Grade 3	N/A
6 (7)	71	M	NSCLC	N	Nivolumab 240 mg every 2 weeks	12 (290)	Grade 1	N/A
7 (16)	68	M	squamous lung cancer	N	Sintilimab 200 mg + PTX liposomes/CBP every 3 weeks	3 (46)	Grade 4	N
8 (17)	78	M	Squamous lung cancer	Colon cancer, diabetes	Pembrolizumab 200 mg + nab-paclitaxel/nab-paclitaxel (2nd cycle) every 3 weeks	2 (44)	Grade 4	N/A
9 (18)	73	F	Lung adenocarcinoma	N	Nivolumab at 3 mg/kg every 2 weeks, third-line treatment	186 days	Grade 4	Grade 4 hypothyroidism, grade 3 anemia
10 (18)	70	M	Lung adenocarcinoma	N	Nivolumab at 3 mg/kg every 2 weeks, third-line treatment	162 days	Grade 3	Grade 4 hypothyroidism, grade 3 anemia
11 (18)	78	M	Lung adenocarcinoma	N	Nivolumab at 3 mg/kg every 2 weeks, 2nd-line treatment	1 (15)	Grade 4	Grade 4 hypothyroidism, grade 3 anemia
12 (19)	73	M	Lung adenocarcinoma	COPD; Crohn’s disease	Nivolumab 3 mg/kg every 2 weeks, third-line treatment	5 (90)	Grade 4	N
13 (19)	74	M	Lung adenocarcinoma	Lymphoma	Nivolumab 3 mg/kg every 2 weeks	9 (112)	Grade 4	N
14 (20)	74	F	Lung adenocarcinoma	Ulcerative colitis invasive ductal carcinoma (right breast)	Nivolumab at 3 mg/kg every 2 weeks, 2nd-line treatment	1 (14)	Grade 4	Hepatitis
15 (21)	56	M	Lung adenocarcinoma	N	Nivolumab at 3 mg/kg every 2 weeks, 2nd-line treatment	3 (87)	Grade 4	Grade 4 hypothyroidism, grade 2 anemia
16 (22)	73	F	Lung adenocarcinoma	Autoimmune myositis; Crohn‘s disease; hypothyroidism	Pembrolizumab (not documented)	2 (42)	Grade 4	N
17 (23)	72	M	NSCLC	N	Pembrolizumab 200 mg +Pem/CBP every 4 weeks	4 (118)	Grade 4	Grade 2 hypothyroidism, pneumonitis (26); grade 4 hepatitis (92)
18 (24)	68	M	Non-keratinizing squamous lung carcinoma	IgG monoclonal gammopathy of undetermined significance (MGUS)	Pembrolizumab 200 mg every 3 weeks	1 (10)	Grade 4	Mucositis (grade 2), anemia (grade 1), thrombocytopenia (grade 2)
19 (25)	74	M	Recurrent lung adenocarcinoma	N	Pembrolizumab 200 mg every 3 weeks	4 (16 days after last ICPi)	Grade 4	N
20 (26)	57	M	Lung adenocarcinoma	Pulmonary emphysema	Nivolumab therapy (3 mg/kg every 2 weeks, third-line treatment)	3(29)	Grade 3	Hepatitis (grade 2)
21 (27)	79	M	Recurrent lung adenocarcinoma	N	4 cycles Atezolizumab (1,200 mg) + Bevacizumab /PTX/CBP every 3 weeks maintenance therapy Atezolizumab + Bevacizumab	5 (30 days after last ICPi)	Grade 4	Thrombocytopenia (grade 4)
22 (28)	83	M	Lung non-small-cell carcinoma	COPD, splenomegaly (splenectomy)	Pembrolizumab 200 mg monotherapy	5 (21 days after last ICPi)	Grade 4	N
23 (29)	75	M	Metastatic lung squamous carcinoma	N	Tislelizumab 200 mg + nab-paclitaxel/CBP	1 (4)	Grade 4	Toxic Epidermal Necrolysis (day 13)
24 (30)	67	M	Lung squamous cell Carcinoma	N	Nivolumab therapy (3 mg/kg every 2 weeks, third-line treatment)	35 (not available)	Grade 4	Thrombocytopenia (grade 2)
25 (31)	63	M	Lung squamous carcinoma	N	Nivolumab therapy (2^nd^-line treatment)	1 (24)	Grade 4	Pneumonia (grade unknown)
26 (Our case 1)	68	M	Lung squamous cell carcinoma	Seborrheic dermatitis	Tislelizumab 200 mg+ CBP/PTX liposome every 3 weeks	3 (144)	Grade 4	Hepatitis (grade 2); Thrombocytopenia (grade 4); Y anemia (grade 3)
27 (Our case 2)	73	M	Lung adenocarcinoma	Hypertension	Tislelizumab 200 mg+ Pem/CBP every 3 weeks	2 (32)	Grade 4	Renal injury (grade 2); diarrhea and oral ulceration, thrombocytopenia (grade 3); anemia (grade 3)
**Autoantibody**	**Bone marrow biopsy findings**	**Treatment**			**Outcome**			
		**Corticosteroids**	**G-CSF**	**IVIG**	**Neutropenia recovery**	**Duration of neutropenia(days)**	**Death**	**Neutropenia-related death**
N/A	Granulocyte maturation blockade	N	Y	N	Y	N	N	N
N/A	Normal	N	N	N	N	N	N	N
N/A	All lineage hypoplasia, activated CD8 (+)	N	N	Y	N	N	Y	Y
N/A	All lineage hypoplasia, activated CD8 (+)	Prednisolone (1 mg/kg)	Y	Y	N	N	N	N
N/A	All lineage hypoplasia	Prednisolone (1 mg/kg)	Y	N	N	N	N	N
N/A	Normal	N	N	N	Y	N	N	N
ANA(+) anti-neutrophil: N/A	N/A	Methylprednisolone (200 mg/d)	Y	Y	Y	9	N	N
ANA (1:100; +) anti-neutrophil: N/A	Hypocellular marrow, megakaryocytes spared	Methylprednisolone (200 mg/d)	Y	N	Y	65	Y	N
Antiglobulin tests and ANA negative; anti-neutrophil: N/A	Hypocellular bone marrow, activated CD8 (+) T-cells	N	N	Y	No response to IVIG	30 days withANC < 500	N	Y
Antiglobulin tests and ANA negative; anti-neutrophil: N/A	Hypocellular bone marrow	Prednisolone (1 mg/kg)	Y	N	Y	91 days with ANC < 500	N	N
Antiglobulin tests and ANA negative; anti-neutrophil: N/A	Hypoplastic, erythroblastic bone marrow with dyserythropoiesis and dysgranulopoiesis.	Prednisolone (1 mg/kg)	Y	Y	N	73 days and still ongoing with ANC < 500	Y	N
N/A	ND	Methylprednisolone (1 mg/kg)	Y	N	N	19	Y	Y
ANA, anti-neutrophil antibody negative	Granulocyte hyperplasia+,maturation blockade, CD8/CD4 T-cell ratio reversed	Prednisolone (1 mg/kg)	Y	N	Y	7 (multiple relapses in 6 months)	Y	N
Anti-neutrophil: N/A	ND	Prednisone (1.5 mg/kg) methylprednisolone (2–3 mg/kg)	N	Y	No improvement following IVIG, improvement on methylprednisolone	35	N	N
PR3-ANCA (+) anti-neutrophil: N/A	All lineage hypoplasia, lymphocyte infiltration	Methylprednisolone (500 mg/d)	Y	Y	Y	86	Y	N
ANA: speckled > 1:640 anti-neutrophil: N/A	N/A	Prednisone (70 mg qd)	Y	Y	No improvement following IVIG, improvement on 4-days cyclosporine A	13	N	N
Negative (antinuclear, antimitochondrial antibodies)	Granulocyte maturation arrest (myelocyte stage), no evidence of dysplasia	Methylprednisolone pulse (2,000 mg/day for 3 days)	Y	Y	Y	12	N	N
Negative (ANCA) anti-neutrophil: N/A	Normocellular marrow, absent granulocytic series, discrete plasmacytos (7% of plasma cells)	Methylprednisolone (40 mg/d)	Y	Y	N	20	Y	Y
Negative (antinuclear antibody, ANA, ANCA, RF)	Mildly hypercellular, unremarkable erythropoiesis and megakaryopoiesis, hyperplasia of myelocytic precursors	Prednisolone (1 mg/kg)	Y	N	Y	2 (developed multiple relapses)	N	N
Negative (antinuclear antibody)	Agranulocytosis, no malignant tumor invasion	Prednisolone (0.5–2 mg/kg)	Y	N	Y	13	N	N
Negative (antinuclear antibody)	Myeloid precursors decreased; erythroid/megakaryocytes increased	N	Y	N	Y	33	N	N
Negative (antinuclear antibody, ANCA, RF)	N/A	Prednisolone (125 mg/d)	Y	N	Y	4	N	N
N/A	Low myeloid hyperplasia, granulocytopenia; 1% hemophagocytosis; T lymphocyte 69.5% (CD4/CD8 0.88)	Dexamethasone 10 mg/m^2^	Y	Y	Y	30	N	N
ANA, ANCA (−); platelet-associated immunoglobulin G (weak+), Coombs tests (+)	Hypoplastic bone marrow, but no dysplastic cells, fibrosis, or increased adipose tissue	Prednisolone (50 mg/d)	Y	N	Y	4	N	N
N/A	N/A	N	Y	N	Y	N/A	N	N
N/A	Severe hypoplasia of hematopoietic tissue; mature lymphocytes increased	Methylprednisolone (40-80mg/d)	Y	N	N	19	Y	Y
N/A	N/A	Methylprednisolone (40–80 mg/d)	Y	N	N	9	Y	Y

F, female; G-CSF, granulocyte colony–stimulating factor; ICPi, immune checkpoint inhibitor; IVIG, intravenous immunoglobulins; M, male; N, no; N/A, not available; Y, yes; PTX, paclitaxel; CBP, carboplatin; Pem, pemetrexed;,COPD, chronic obstructive pulmonary disease; N/A, not applicable; ANA, antinuclear antibody; RF, rheumatoid factor; ANCA, anti-neutrophil cytoplasmic antibody.

However, the mechanisms for irN are still unclear. PD1 was found strongly expressed on CD3^+^, CD4^+^, and CD8^+^T cells in patients with aplastic anemia (32). The PD1/PD-L1 axis should be crucial for preventing immune-mediated damage of the haematopoietic niche. Finkel et al. documented that T-cell activation, autoantibody production, and increase in cytokine secretion have been contribution to the development of irN (33). This meta-analysis (*N* = 32) classified irN into three subtypes: central type (25%) characterized by predominantly CD8^+^ T cell-mediated bone marrow aplasia and poor prognosis; peripheral type (12%) associated with anti-neutrophil antibodies (ANAs) and bone marrow hyperplasia with better prognosis; modified peripheral type (63%) with favorable prognosis but lacking either anti-neutrophil antibodies or bone marrow hyperplasia (29, 33). Limited ANGA testing makes it hard to distinguish peripheral and modified peripheral types, but both responding well to glucocorticoids and IVIG. Delanoy et al. reported 80% (4/5) immune-related pancytopenia cases showed severe trilineage myelohypoplasia on bone marrow biopsy, consistent with a central mechanism (7, 18).

A recent study demonstrated that patients with a history of lymphocytic leukemia and autoimmun disease exhibit an increased incidence of hem-irAEs (7, 34, 35). About 23%–25% of hem-irAEs are complicated with other systemic irAEs (33). In our literature (Table 2), 20 cases (80%) had grade 4 irNs, five cases had positive antibody, six cases (24%) had a prior history of autoimmune disease or hematologic disorders, including Crohn’s disease, ulcerative colitis, autoimmune myositis, lymphoma, IgG monoclonal gammopathy of undetermined significance, and splenomegaly (19, 20, 22, 24, 26), and 5 cases (20%) were complicated with other organ-related irAEs, including hepatitis, renal injury, mucositis, pneumonia, and Toxic Epidermal Necrolysis (20, 23, 24, 26, 29). In our two cases, the patients did not have any autoimmune disease diseases history or lymphoma, but one patient had the history of seborrheic dermatitis and pulmonary interstitial changes with 51.26% of diffusing capacity of the lung for carbon monoxide per unit alveolar volume (DLCO/VA). Thus, more detailed mechanisms of hem-irAEs need to be further explored.

Confounded by underlying diseases and prior therapies, irN is challenging to diagnose, especially when complicated by chemotherapy and severe infection. Compared to the absolute neutrophil count changes in chemotherapy-related neutropenia follow a U-shaped trend approximately 7–14 days after chemotherapy (36, 37), the irN can manifest at any time (8). The median onset of neutropenia in patients treated with PD-1/PD-L1 antibodies was 10.5 weeks after the first administration of ICI (2.2–25.4 weeks) (38). Kramer et al. documented the median time of occurrence for hem-irAE in patients treated with immunochemotherapy was earlier than who were treated with PD-1/PD-L1 monotherapies (12 vs. 25 weeks) (12). Pemetrexed typically causes grade 3–4 neutropenia in 5%–15% of patients with a delayed nadir, slow decline, and gradual recovery upon discontinuation. In contrast, our cases showed abrupt neutropenia onset 4.6–20.6 weeks (32–144 days) after initial tislelizumab exposure, with no response to treatment, suggesting Hem-irAE.

Infection is one of the most fatal complications in patients with irN. Severe infection can induce pancytopenia via mechanisms including direct bone marrow damage by pathogens and histiocytic hemophagocytosis (39). This condition can be caused by *Mycobacterium tuberculosis*, *Salmonella typhi*, *Brucella* and other pathogens, while fungal infection is rarer and only occurs in disseminated systemic infection. Most infection-associated myelosuppression is transient, with marrow function recovering after infection control (40). In addition, long-term use of broad-spectrum antibiotics impairs bone marrow hematopoietic function, mainly attributed to intestinal flora disturbance, bone marrow regulatory T cell depletion and disrupted hematopoietic microenvironment (41, 42). Lung cancer with bone marrow metastasis is rare, has an extremely poor prognosis, is more common in small cell lung cancer and often accompanied by bone metastasis (43, 44). Asymptomatic in the early stage, it presents bone pain, fractures and pancytopenia in the advanced stage, with bone marrow biopsy showing malignant tumor infiltration as the gold standard. Wang et al. (44) summarized 12 Chinese confirmed cases, 10 with bone metastasis, and isolated anemia was the most common hematological change (5/12, 41.7%).

Grade 4 neutropenia developed on Day 144 after Cycle 1 in Case 1, well beyond the typical nadir of cytotoxic drug-related myelosuppression. Bone marrow examination revealed severe hypoplasia and marked lymphocytic infiltration, without malignant cells or pathogens. These findings strongly support an immune - mediated central mechanism as the primary cause. Infection - associated myelosuppression was only a concomitant complication. Although the second patient declined BMB, we also speculated the primary cause of pancytopenia is hem-irAEs basing on the following points: (1) The onset and the persistently unrelieved course are more consistent with hematological irAEs. (2) We classified the adverse events using the Naranjo scale, rating them as “possible.” (3) Systemic infection developed on Day 34 after grade 4 neutropenia and without bone metastasis or pain. Pancytopenia secondary to infection or marrow metastasis was excluded. (4) Hem-irAEs are typically the most common type of concomitant toxicity (33). The patient also developed immune-related renal injury, mucositis and gastrointestinal toxicity, while Case 1 was complicated with hepatitis (Table 1).

No consensus exists on managing immunotherapy-related hematologic toxicity (3, 6, 10). Reported treatments for hem-irAEs include corticosteroids, G-CSF, TPO, IVIG and blood transfusion. Rituximab and tumor necrosis factor antagonists are options for refractory cases, while cyclosporine and androgens can be used for severe aplastic anemia (7, 13, 45). Glucocorticoids are first-line treatment with a 20% failure rate. Grade 3/4 irAEs require immediate ICI discontinuation and timely intervention. Optimal steroid initiation timing and treatment duration remain unclear in guidelines. Of the 25 patients listed in Tables 2, 10 cases were applied combination therapy of corticosteroids and G-CSF, four patients had responds on neutropenia; eight cases received combination therapy of corticosteroids, G-CSF and IVIG, the irN in four patients were resolved. In contrast, IVIG monotherapy demonstrated limited efficacy in achieving disease remission in two additional patients (7, 18). In our presentation, methylprednisolone (0.36–0.82 mg/kg/d) was administered within 1–4 days of neutropenia and/or thrombocytopenia onset when initial treatments proved ineffective, and gradually increase to 1.44–1.64 mg/kg/d. The clinical course lasted between 9 and 33 days prior to the patient’s demise. The fatality was primarily attributed to severe treatment-related complications, which may have partly resulted from the delayed initiation of corticosteroid therapy and an insufficient dose, a poor therapeutic response to corticosteroids, or the lack of combination with other immunosuppressive therapies.

Several limitations exist in this study. First, before initiating ICI, we screened for viral hepatitis, HIV, COVID-19, T-SPOT, and fungal infection, and the results were normal. However, we omitted autoantibodies, including ANA, ANCA, and ANGA etc. Second, bone marrow biopsy was not performed in Case 2, and neither patient received additional therapies including IVIG, rituximab or cyclosporine due to financial constraints and limited clinical experience. Third, full-cycle hematological follow-up was not well-performed. Fourth, our study has a selection bias toward severe cases, which may overestimate the severity of hem-irAEs.

In conclusion, tislelizumab combined with chemotherapy may induce pancytopenia, particularly persistent grade 4 neutropenia. Though rare, it can result in fatal infections. Early glucocorticoid therapy is indicated for suspected hem-irAEs that are unresponsive to routine treatment or complicated with other organ dysfunction. Central-type pancytopenia is often steroid-resistant. Therefore, timely bone marrow biopsy and adjuvant IVIG, rituximab, or cyclosporine are recommended. This study supplements clinical evidence for hem-irAE management. Further research is required to elucidate its pathogenesis and facilitate early diagnosis.

## Data Availability

The original contributions presented in this study are included in this article/supplementary material, further inquiries can be directed to the corresponding author.
